# Improving the sustainability of the wheat supply chain through multi-stakeholder engagement

**DOI:** 10.1016/j.jclepro.2021.128837

**Published:** 2021-10-25

**Authors:** Lijuan Deng, Hongyan Zhang, Chong Wang, Wenqi Ma, Annah Zhu, Fusuo Zhang, Xiaoqiang Jiao

**Affiliations:** a National Academy of Agriculture Green Development, Department of Plant Nutrition, China Agricultural University, Beijing, 100193, PR China; b College of Resources and Environment Science, Hebei Agricultural University, Baoding, 071001, Hebei, PR China; c Environmental Policy Group, Wageningen University, Wageningen, 6708 LX, Netherlands

**Keywords:** Agricultural sustainability, Food supply chain, Smallholder production, Wheat

## Abstract

Feeding the world's growing population, while producing economic benefits with limited environmental effects, is a major challenge faced by global food supply chains. This is especially apparent when the production stage is predominated by smallholders as they each face varying economic and environmental demands, making it difficult to mobilize them on the ground. This study investigated how the environmental and economic sustainability of wheat supply chains could be improved by analyzing the performance of all stakeholders, especially the smallholders. Results showed that 77% of GHG emissions came from wheat cultivation, and less than 8% of the total economic benefits were recouped during this stage. In contrast, smallholders in the Science and Technology Backyards, reduced their GHG emissions by 16.4% and improved their economic benefits by 1.3- fold. Furthermore, a 2.6-fold increase in profit (1808 USD) with GHG emission reduction was achieved simultaneously by integrating all individual stages as a whole. This study found that the sustainability of the wheat supply chain was mainly affected by wheat cultivation. It also demonstrated the potential efficacy of empowering smallholders and integration of all individual stages as a whole to improve the sustainability of food supply chains.

## Highlights

• A complete supply chain analysis for steamed bread was undertaken.
• Wheat planting is the major limiting stage for sustainability of wheat supply chain.
• Approaches to improve food supply chain sustainability have been developed.

## Graphical abstract

**Figure:**
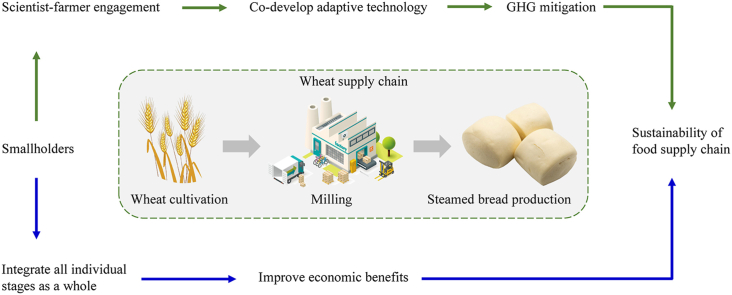

## 1 Introduction

The global food supply chain will face significant challenges in the 21st century. To meet the needs of a growing population by 2050, it will need to provide more food and reduce its effect on the environment (Tilman et al., 2011). Such challenges are especially daunting in developing countries, due to their rapidly growing populations and changing diets (Huang and Yang, 2017). The food supply chain is a complex entity, linking producers, processors, markets, and distributors (Osei-Owusu et al., 2019). However, in rapidly developing countries, the components of the supply chain are highly fragmented (Roth et al., 2008). This fragmentation has caused repercussions such as low N use efficiency (NUE) and high environmental risk, contributing to diminishing profits, and hence, the reduced sustainability of the world's food supply chain (Jiao et al., 2019). Overcoming this fragmentation through integrated action, engaging all stakeholders (e.g., crop producers, food processors, marketers, and distributers) and stimulating their contribution potential is the key to its sustainability.

The food supply chain links production and consumption, encompassing environmental and economic domains (Blasi et al., 2016). Its overall performance depends on each individual stage and respective processes (Acquaye et al., 2011; Garnett, 2011). For example, crop production is responsible for the majority of anthropogenic environmental effects (Kulak et al., 2015), and in the UK wheat-to-bread supply chain, it was found that most greenhouse gas (GHG) emissions were primarily attributable to wheat production, with 56% coming from synthetic N fertilizer use (Goucher et al., 2017). This will increase environmental risks related to N loss (ammonia volatilization etc.) (Ju et al., 2009; Vitousek et al., 2009). Moreover, based on 38,700 commercially viable farms in 119 countries across 40 products, farming activities accounted for 61% of the GHG emissions due fertilizer use in crop production (Kulak et al., 2015). Therefore, improving NUE in crop production is an effective approach for achieving sustainability.

A series of technological advancements have been developed to improve NUE in crop production (MacMillan and Benton, 2014; Zhang et al., 2016). For instance, based on field trials in the North China Plain, NUE in maize production could be improved by 100% with an integrated soil–crop system management approach (Chen et al., 2011). From field trials in Pakistan, optimum N use was found to be important for attaining higher NUE, potentially enhancing sunflower growth and yield (Nasim et al., 2018). Similarly, optimizing chemical fertilizers to meet crop demand is an effective approach to reduce environmental effects (Klammsteiner et al., 2020). However, such results have only been obtained from experimental field plots under precise management conditions. Globally, wheat production is primarily dominated by smallholders that can lack in effective information and technical support (Fenu and Malloci, 2020). As primary material suppliers within the food supply chain, their capabilities should be improved to reduce their environmental effect. However, approaches on how to achieve this have not been fully investigated.

Another major limiting factor for the sustainability of the food supply chain is the lack of economic benefits (Chinseu et al., 2019). In most developing countries the food supply chain acts as the primary income generator for stakeholders at each stage (Yang, 2006); thus, economic benefits are a major motivating factor (Yang, 2006). Previous studies have shown that economic and environmental effects could be addressed at the same time by smallholders changing their cropping patterns (Hashemi et al., 2019). Given that smallholders provide primary material, integrating them into the food supply chain and strengthening their farming capacity is an effective approach to improve the competitiveness of the supply chain as a whole (Dokić et al., 2020). However, potential approaches to improve the sustainability of the food supply chain by balancing economic benefits and environment risks on the ground have not been adequately investigated.

Building on the need for a full supply chain analysis, we tested the hypothesis that food supply chain sustainability could be improved by empowering smallholders through scientist–farmer engagement during the wheat production stage and by integrating all individual stages as a whole. In this case study of the wheat supply chain involving smallholders, the objectives were to: (1) investigate NUE, GHG emissions, and economic benefits at each stage in the supply chain to identify the key limiting components of sustainable development of the whole chain; (2) examine potential adaptive management strategies in smallholder farmer practices (i.e., the Science and Technology Backyards [STB] method) and their effects on NUE, GHG emissions, and economic benefits; and (3) suggest pathways forward to maximize economic benefits and reduce environmental risks of the food supply chain by improving NUE.

## 2 Materials and methods

### 2.1 System boundaries

The study area is located in Quzhou County, Hebei Province, in the North China Plain where with annual rainfall of 450–550 mm, of which 30% occurs during the wheat growing season. Annual average temperatures range from 11 to 14 °C with a frost-free period of about 210 d. Wheat is a major staple crop in the North China Plain, accounting for ∼56% of national wheat production (Du et al., 2014). Most of the wheat is planted in winter with an average yield of 6.7 t ha^−1^ in 2018 (Jiang et al., 2020).

**Fig. 1:**
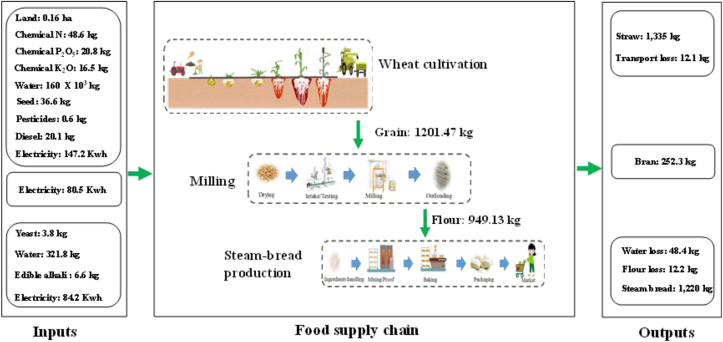
Flow of raw materials throughout the wheat supply chain. Values represent the quantity of inputs and outputs needed to meet the daily consumption needs of 10,000 people (1220 kg of steamed bread per day). The demand for steamed bread on the North China Plain was assumed to be 0.12 kg person^−1^ day^−1^ (CNS, 2013).

In this study, we categorized the wheat supply chain according to three distinct components: wheat cultivation, flour processing, and steamed bread production (Fig. S1). Primary data was collected for each of these components in 2018. The distribution of each component is illustrated in Fig. 1, S1 and S2 (primary data indicator details are listed in Table S1). The flowchart of research methodology was presented in Figure S3. During wheat cultivation, data was collected by three techniques: farmer surveys (n = 265 farmers, those who carried out conventional farmer practices, or “FP”), farmer monitoring (n = 59 farmers engaged in STB programs, hereafter “STB farmers”), and long-term field experiments under optimal management conditions (hereafter, “OPT” scenarios). The survey was conducted on a 1 km grid covering the whole of Quzhou County. Indicators collected included wheat yield, chemical fertilizer use, land use, irrigation, harvest, labor, and income. For the farmer monitoring, the STB farmers were those willing to participate in technology innovation and knowledge transfer through STBs established in Wangzhuang Village.

Compared with FP, the practices by STB farmers included participation in scientists’ research, resulting in co-developed adaptive technologies for improved yield and sustainability, including optimal chemical N supply intensity, modification of seeding rate, and zinc biofortification. Concurrently, the OPT scenarios comprised experiments on high wheat yield and high NUE conducted by scientists at the Quzhou Experiment Station of China Agricultural University (36°52′ N, 115°02′ E). In addition to the adaptive technologies employed by STB farmers, OPT farms also used optimal N topdressing.

For the later supply chain stages, flour processing and steamed bread production data was obtained from 20 manufacturers (10 for each stage in each town). For FP, smallholders sold the wheat to manufacturers without being directly involved in the later supply chain stages. For STB farmers, 59 smallholders established a farmer cooperative, supported by STB, thereby generating the capacity for sustainability at all stages.

For the OPT scenarios, further connections were made to better integrate cultivation and later production stages, including whole-meal bread production and the scientist–smallholder engagement. Wheat production was cultivated based on the specific demand of the later stages from a larger company and the related wheat grain was purchased by that company.

### 2.2 Substance flow analysis (SFA)

A dynamic partial substance flow analysis (SFA) model was developed to quantify N flow in the system. Based on mass balance, the model used the following calculation: input equals output plus stock for the total system. Inputs included N from chemical fertilizer, irrigation, deposition, seed, and biological fixation in wheat production, and N from yeast in steamed bread production. Outputs included the steamed bread and its byproducts (wheat bran). Stock represented the difference between input and output, including N loss in wheat production (denitrification, leaching, and runoff, N accumulation in arable land, and ammonia [NH_3_] volatilization), N loss in grain transport to flour manufacture, and N loss from low-quality wheat and steamed bread production.

### 2.3 N losses

In this study, N losses, N accumulation, and NUE in the system were quantified. Total N loss included the loss of nitrous oxide-N (N_2_O-N), nitrate-N (NO_3_-N), and ammonia-N (NH_3_-N) from wheat production, as well as transport losses, low-quality wheat loss, and flour loss during milling and steamed bread production. N loss in wheat production was estimated from the N application rate and N surplus according to empirical models from the North China Plain (Chen et al., 2014):

(1) $${\text{N}}_{\text{NH}3}\;=\text{ 0.17 × N }-\;4.95\text{,}$$

(2) $${\text{N}}_{\text{NO}3}\;=\;13.59\text{ × }{\text{e}}^{\left(0.009\text{ × S}\right)}\text{,}$$

(3) $${\text{N}}_{\text{N}2\text{O}}\;=\;0.54\text{ × }{\text{e}}^{\left(0.0063\text{ × S}\right)}\text{,}$$

(4) $$\text{S }=\;{\text{N}}_{\text{rate}}\;-\;{\text{N}}_{\text{uptake}}$$

where S is the N surplus, which is defined here as chemical N fertilizer use minus N removal at harvest. The N removal at harvest was estimated from the relationship between wheat N removal and grain yield:

(5) $${\text{N}}_{\text{uptake}}\;=\;-\text{14 }+\;41\text{ × }{\text{Y}}^{0.77}$$

where N_uptake_ is the N uptake in the wheat and Y is the grain yield. Please see the detailed methodology from previous studies (Chen et al., 2014; Ying et al., 2019).

N loss from straw was estimated as follows:

(6) $${\text{N}}_{\text{s}}\;=\;{\text{N}}_{\text{straw}}\text{ × }{\text{M}}_{\text{straw}}$$

where N_s_ is the N loss from straw, N_straw_ is N concentration of wheat straw, and M_straw_ is the biomass of straw lost from arable land.

For N loss from transport, low-quality wheat grain and flour loss in steamed bread production was determined for the manufacturers. N loss was estimated as follows:

(7) $${\text{N}}_{\text{t}}\;=\;{\text{N}}_{\text{c}}\text{ × }{\text{M}}_{\text{transport}}$$

(8) $${\text{N}}_{\text{f}}\;=\;{\text{N}}_{\text{f}}\text{ × }{\text{M}}_{\text{flour}}$$

(9) $${\text{N}}_{\text{low}}\;=\;{\text{N}}_{\text{c}}\text{ × }{\text{M}}_{\text{low}}$$

where N_t_, N_f_, and N_low_ are the N losses from transport, flour losses, and low-quality wheat, respectively; and M_transport_, M_flour_, and M_low_ are the amounts of loss from transport, flour losses, and low-quality wheat, respectively.

### 2.4 N accumulation

N balance in arable land was estimated as the difference between the N input and output. The N balance is composed of N accumulation and straw that are stored in the field. Thus, the N content of the soil accumulation could be calculated according to mass balance, as follows:

(10) $${\text{N}}_{\text{accum}}\;=\;{\text{N}}_{\text{input}}\;-\;{\text{N}}_{\text{output}}\;-\;{\text{N}}_{\text{loss}}$$

where N_input_ is the total input of N (the sum of N_irr_, N_rate_, N_deposition_, N_seed_, and N_BF_ in kg), N_output_ is the total output of N (the sum of N_-bread_ and N_bran_ in kg), and N_loss_ is the total loss of N (the sum of N_NH3_, N_NO3_, N_N2O_, N_s_, N_t_, N_f_, and N_low_ in kg).

### 2.5 NUE

NUE was calculated as the ratio between total N output in the finished steamed bread product and its byproducts (bran) over the total N inputs in the system:

(11) $$\text{NUE }=\;{\text{N}}_{\text{output}}{\text{/N}}_{\text{input}}$$

### 2.6 Global warming potential (GWP)

A life cycle assessment (LCA) approach involving the global warming potential (GWP) was used to evaluate the environmental effects of the wheat-to-bread supply chain. The functional unit was defined as the total GWP (expressed as kg of carbon dioxide equivalents [kg CO_2_eq]) for meeting the daily steamed bread consumption needs of 10,000 people (equivalent to 1220 kg of steamed bread per day) (China Nutrition Society , 2013). The system boundaries were set as “cradle to grave,” meaning “from field to consumer” when applied to food supply chains. The LCA inventory had two parts: emissions from agriculture and manufacturing inputs, and emissions from production in the field. All emission factors were obtained from Chen et al. (2014).

The leaching, runoff, and volatilization of nitrogenous compounds (such as NH_3_ and NOx, with subsequent redeposition) in wheat cultivation were estimated using the model of Chen et al. (2014). The indirect N_2_O emissions were estimated using IPCC (Intergovernmental Panel on Climate Change) methodology, in which 1% of the volatilized NH_3_-N and 0.75% of leached NO_3_-N is lost as N_2_O-N. Putting this together, the total GHG emissions for meeting 10,000 people's steamed bread consumption was calculated as follows:

(12) $$\text{GHG }=\;\left({\text{GH}}_{\text{Gm}}\;+\;{\text{GH}}_{\text{Gt}}\;\right)\text{× }{\text{N}}_{\text{fert}}\;+\;{\text{N}}_{\text{N}2\text{O}}\text{ × }44\text{/}28\text{ × }298\;+\;{\text{GHG}}_{\text{others}}\;+\;{\text{GHG}}_{\text{electri}}\;+\;{\text{GHG}}_{\text{yeast}}$$

where GHG_m_ and GHG_t_ are the GHG emissions from chemical N manufacture and transportation per unit of chemical N fertilizer, respectively (expressed as kg CO_2_eq kg^−1^ N); GHG_others_ is the GHG emissions from chemical phosphorus (P) and potassium (K) fertilizer, pesticides, herbicides, diesel consumption for irrigation, land preparation, and harvest in wheat production, including their production inputs, transportation, and application; GHG_electri_ is the GHG emissions due to electricity consumption during milling and steamed bread production; and GHG_yeast_ is the GHG emissions due to yeast consumption in steamed bread production.

### 2.7 Benefit–cost analysis

A benefit–cost analysis was performed to evaluate the cost and profits in the wheat supply chain. The prices of inputs were set to the Quzhou County 3-year average. The costs of the system were calculated as follows:

(13) $${\text{T}}_{\text{cost}}\;=\;{\text{I}}_{\text{land}}\text{ × }{\text{P}}_{\text{land}}+\;{\text{I}}_{\text{electricity}}\text{ × }{\text{P}}_{\text{electricit y}}{+\text{I}}_{\text{pesticides}}\text{ × }{\text{P}}_{\text{pesticides}}\;+\;{\text{I}}_{\text{seed}}\text{ × }{\text{P}}_{\text{seed}}\;+\;{\text{I}}_{\text{N}}\text{ × }{\text{P}}_{\text{N}}\;+\;{\text{I}}_{\text{P}}\text{ × }{\text{P}}_{\text{P}}\;+\;{\text{I}}_{\text{k}}\text{ × }{\text{P}}_{\text{K}}\;+\;{\text{I}}_{\text{Zn}}\text{ × }{\text{P}}_{\text{Zn}}\;+\;{\text{I}}_{\text{diesel}}\text{ × }{\text{P}}_{\text{diesel}}\;+\;{\text{I}}_{\text{yeast}}\text{ × }{\text{P}}_{\text{yeast}}\;+\;{\text{I}}_{\text{water}}\text{ × }{\text{P}}_{\text{water}}\;+\;{\text{I}}_{\text{edib}}\;+\;{\text{P}}_{\text{deib}}$$

where I_i_ is the input for the food supply chain and P_i_ is the unit price of the input, and:

(14) $${\text{T}}_{\text{benefit}}\;=\;{\text{O}}_{-\text{bread}}\text{ × }{\text{P}}_{-\text{bread }}+\;{\text{O}}_{\text{bran}}\text{ × }{\text{P}}_{\text{bran}}$$

where O_-bread_ is the amount of steamed bread production, and P_steamed-bread_ is the unit price of steamed bread.

The benefit–cost ratio (BCR) was calculated as follows:

(15) $$\text{BCR }=\;{\text{T}}_{\text{benefit}}{\text{/T}}_{\text{cost}}$$

## 3 Results

### 3.1 Raw materials and N flow throughout the supply chain

A total of ∼0.16 ha of arable land was required to meet the daily consumption needs of 10,000 people (1220 kg of steamed bread per day) (Fig. 1). This amount of land produced 1214 kg of wheat grain using the following inputs: 48.6 kg N (304 kg ha^−1^), 20.8 kg phosphorus oxide (P_2_O_5_) (130 kg ha^−1^), 16.5 kg potassium oxide (K_2_O) (103 kg ha^−1^), 36.6 kg seed (229 kg seed ha^−1^), 160 m^3^ water (1000 m^3^ ha^−1^), 147 kWh electricity for irrigation (920 kWh ha^−1^), 0.57 kg pesticide (3.57 kg ha^−1^), and 20.1 kg diesel for land preparation and harvest (126 kg ha^−1^) (Fig. 1). When transferred to the mill, two key output streams were identified. First, ∼1% of wheat grain was lost due to high moisture or the presence of wheat awn due to low-quality or transport loss. Second, ∼21% of wheat grain was separated out as wheat bran and not used for bread production. Further, during the milling, a total of 949 kg of flour was produced with 80.5 kWh of electricity (Fig. 1). Before steamed bread production, a number of ingredients, including 3.8 kg of yeast, 6.64 kg of edible alkali, and 322 kg of water, was added into the flour to produce the 1220 kg of steamed bread. In the process, 12.2 kg of flour was lost due to suboptimal quality and 48.4 kg of water was lost due to cooling. The total electricity consumed during steamed bread production was 84.2 kWh (Fig. 1).

**Fig. 2:**
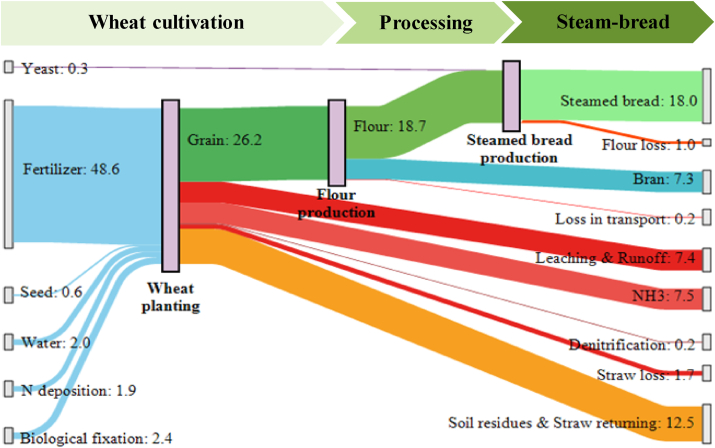
Nitrogen flow (kg) in the wheat to steam bread supply chain. Values represent the quantity of inputs and outputs needed to meet the daily consumption needs of 10,000 people (1220 kg of steamed bread per day). The demand for steamed bread on the North China Plain was assumed to be 0.12 kg person^−1^ day^−1^ (CNS, 2013).

N flow along the wheat supply chain was quantified through SFA (Fig. 2), finding that growing enough wheat to produce 1220 kg of steamed bread on 0.16 ha of arable land requires 48.6 kg N in chemical fertilizer, 0.6 kg N in seed, 2 kg N in irrigation water, 1.9 kg of N deposition, and 2.4 kg N from biological fixation. During wheat production, 26 kg N was used to produce wheat grain and a total of 29.3 kg of N was lost to the environment: 7.5 kg N lost as NH_3_, 0.2 kg N lost due to denitrification, 7.4 kg N lost as leaching and runoff, up to 12.5 kg N accumulated in the soil and 1.7 kg N lost as straw removal. During flour production, three key N output streams were identified: 18.7 kg N as flour, 7.3 kg N as byproduct and 0.2 kg N as low-quality wheat. Before steamed bread production, 0.3 kg N was added in the form of yeast and 1.0 kg N was lost as low-quality flour. The final preparation and steaming process resulted in 18.0 kg N for the steamed bread produced.

### 3.2 NUE and loss based on different farming practices

**Fig. 3:**
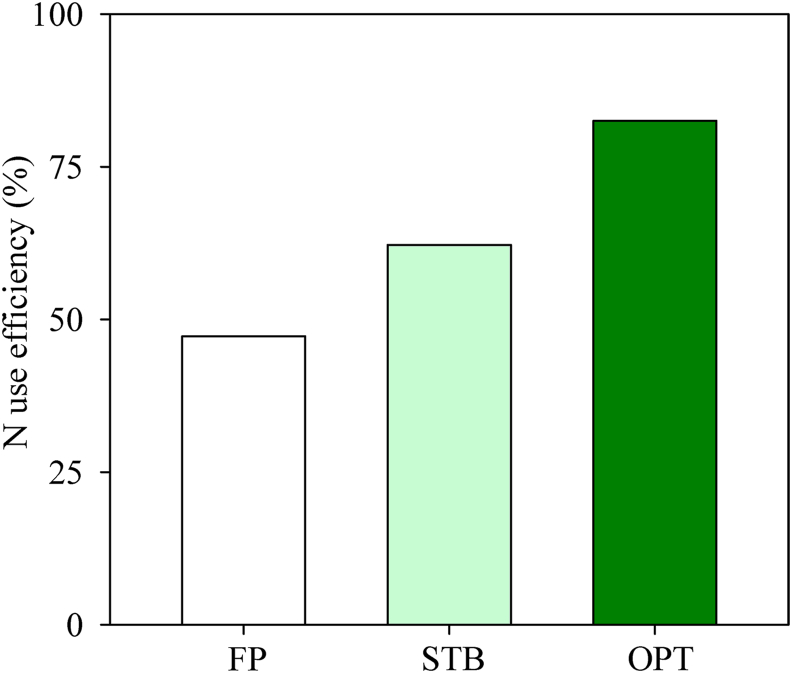
Nitrogen use efficiency (ratio of N input to N output) for the wheat supply chain under different groups: typical farmer practices in the region (FP), farmers engaged in STB programs (STB), and optimal solutions based on experimental field testing (OPT). Values were calculated based on the daily consumption needs of 10,000 people (1220 kg of steamed bread).

**Fig. 4:**
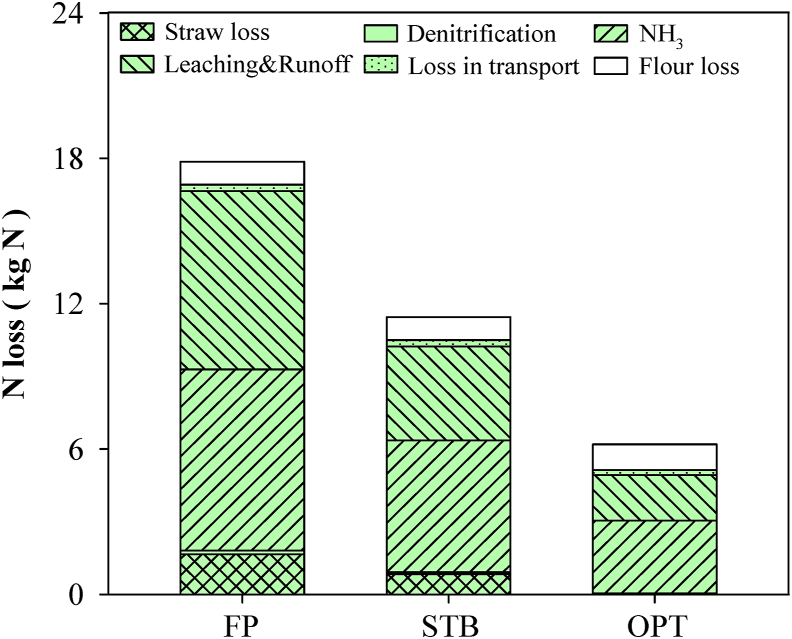
Nitrogen losses for the wheat to steamed bread supply chain under the different groups: FP, STB, and OPT. Values were calculated based on the daily consumption needs of 10,000 people (1220 kg of steamed bread).

For FP, NUE in the wheat supply chain was 47% (Fig. 3). Wheat production was a major contributor to N loss (Fig. 4), and chemical fertilizer used to increase wheat growth was found to be the largest single process contributing to N loss. More than 95% of N loss for FP occurred during wheat production, with the rest occurring during the later stages due to low-quality wheat and flour (Fig. 4). For FP, about 42% of N was lost as NH_3_ volatilization.

Compared with FP, the practices by STB farmers improved NUE to 62% through integrated nutrient management. For example, N application was reduced to 258 kg N ha^−1^ and sowing to 160 kg ha^−1^ to give an appropriate wheat population for high yield. The OPT demonstrated even greater improvements in NUE; N application was reduced to 190 kg N ha^−1^ and wheat yield was improved to 9 t ha^−1^. For OPT scenarios, NUE was improved to 83% and N loss was reduced to 6.2 kg.

### 3.3 Global warming potential based on different farmer practices

**Fig. 5:**
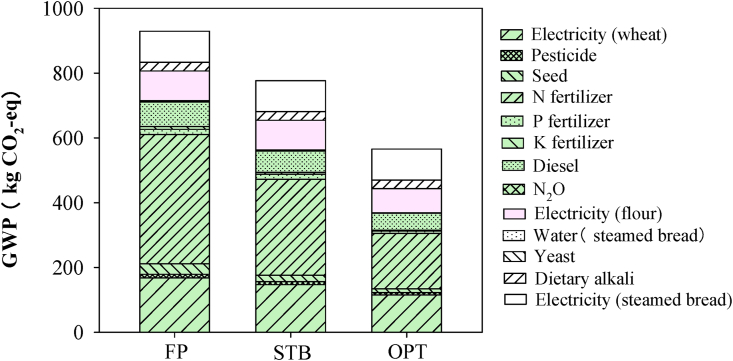
Global warming potential (GWP) for the wheat supply chain under the different groups: FP, STB, and OPT. Values were calculated based on the daily consumption needs of 10,000 people (1220 kg of steamed bread per day).

From the LCA of GWP for each wheat supply chain stage (Fig. 5), the GWP from the whole supply chain was 930 kg CO_2_eq for producing 1220 kg of steamed bread and, wheat production was the main source. For FP, ∼400 kg CO_2_eq was emitted from chemical fertilizer use, and electricity used for irrigation resulted in 168 kg CO_2_eq. Other processes involved in wheat production had a lesser effect than chemical fertilizer use and electricity for irrigation. The consumption of diesel for land preparation and grain harvest accounted for only 10.5% of GWP. Flour processing added another 91.8 kg CO_2_eq and steamed bread production added another 123 kg CO_2_eq.

Compared with FP, the practices by STB farmers reduced GWP to 777 kg CO_2_eq through reduced chemical fertilizer use, especially N consumption, and improved wheat yield. The OPT farm practices further reduced GWP to 566 kg CO_2_eq.

### 3.4 Economic benefits based on different farming practices

For FP, the total cost of producing 1220 kg of steamed bread was 570 USD (Fig. S3). Wheat production was the principal contributor to the total cost (e.g., for land preparation, chemical fertilizer, seed, and land preparation/harvest machinery), accounting for 68.3%. Among all the costs in wheat production, the land preparation cost was the highest (about 56.7%). In addition, the cost of machinery for land preparation and harvest accounted for 12.4%. Flour processing added an additional 7 USD due to electricity consumption, and steamed bread production cost 137 USD for yeast, edible alkali, and electricity.

Based on our analysis, total costs could be reduced by 10.9% with the adaptive technologies used by the STB farmers. For example, compared with FP, practices by STB farmers reduced land costs by 12.5% due to improved wheat yield, while simultaneously also reducing chemical N fertilizer costs by 25.7%. From the analysis of OPT field practices, the cost of land preparation and chemical fertilizer could be reduced by 31.3% and 57.0%, respectively, due to improved wheat grain yield and reduced chemical N input.

**Fig. 6:**
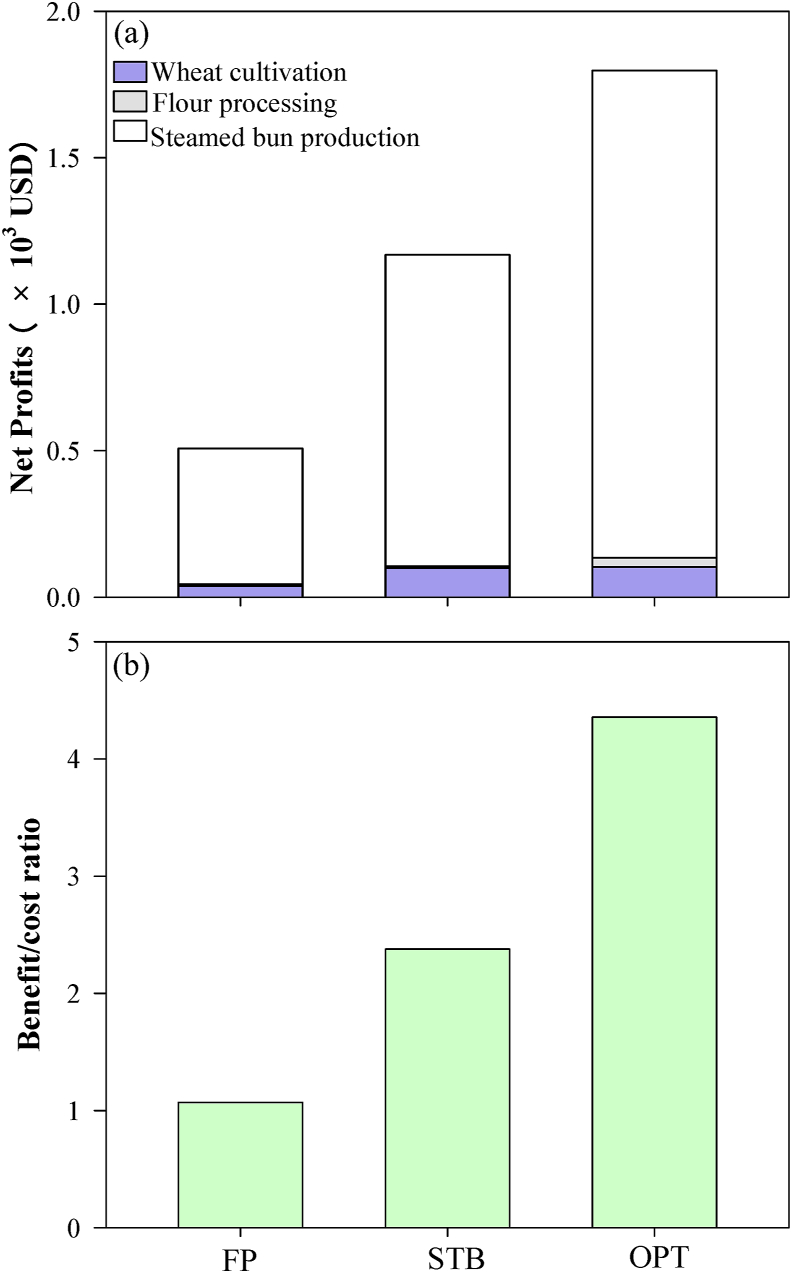
Net profits (a) and benefit–cost ratio (b) for the wheat supply chain under the different groups: FP, STB, and testing OPT. Values were calculated based on the daily consumption needs of 10,000 people (1220 kg of steamed bread per day).

On average, wheat production, flour production, and steamed bread production accounted for 7.6, 1.3, and 91.1% of the total net profits of the wheat supply chain, respectively. The ratio, however, shifted depending on farmer practices. For example, the net profit for FP was only 507 USD (Table S2, Fig. 6), while the practices by STB farmers and OPT generated a net profit of 1169 (2.3-fold) and 1808 USD (3.6-fold), respectively, due to improved wheat yield and reduced chemical fertilizer use. Compared to FP, the BCRs of practices by STB farmers and OPT farms improved by 1.2 and 3.1 times, respectively (Fig. 5b).

### 3.5 Changing smallholder farmer practices

**Fig. 7:**
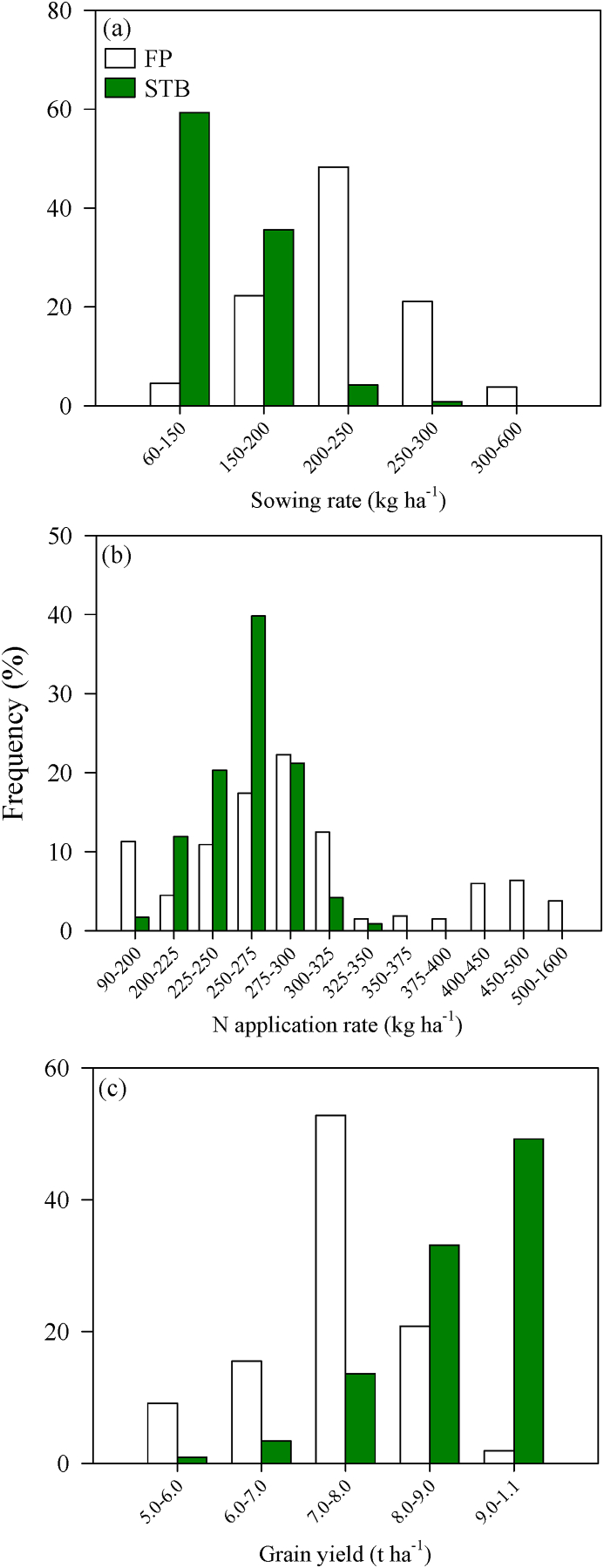
Frequency of sowing rate (a), N application rate (b), and grain yield (c) for practices by STB farmers compared to typical farmer practices (FP).

Compared with FP, the practices of STB farmers changed due to their participation in research and training (Fig. 7). About 48% of FP farmers used 200–250 kg seed ha^−1^, while 95% of STB farmers used less seed, indicating a reduced seeding rate by 30.3%. Chemical fertilizer use by FP farmers was 304 kg ha^−1^, whereas for STB farmers it was 258 kg ha^−1^ (Fig. S4), indicating that ∼74% of STB farmers reduced their chemical fertilizer use. Grain yield for FP farmers was 7.5 t ha^−1^ but was 9 t ha^−1^ for 49% of STB farmers.

### 3.6 Potential effects and scenario analysis

For the whole county, 24.8 × 10^8^ kg of chemical N fertilizer is needed to produce enough steamed bread to service 1395 million people for one year. If all farmers practice adaptive management similar to the STB farmers, 6.4 × 10^8^ kg of chemical N fertilizer could be saved. Similarly, if all farmers could apply the agronomy used by OPT scenarios, 14.1 × 10^8^ kg of chemical N fertilizer could be saved (Fig. 7).

Assuming production of the same amount of steamed bread, net profit in the supply chain of China was found to be 2.6 × 10^4^ million USD under FP. Under the practices of STB farmers and OPT scenarios, profits could be increased to 6.0 × 10^4^ and 9.2 × 10^4^ million USD, respectively. Similarly, GWP in the whole supply chain could be reduced by 16.4% and 39.1%, respectively.

## 4 Discussion

### 4.1 Decoupling GHG emissions and economic benefits by improving NUE

Results show distinctively that although only 7.6% of the total profit was generated during wheat cultivation (Fig. 6), up to 68.3% of the cost was incurred in that stage due to the use of agricultural inputs (such as chemical fertilizers) (Fig. S3). Moreover, up to 77% of GHG emissions produced throughout the entire supply chain were incurred during the wheat cultivation stage (Fig. 5). This suggests that wheat production is the most problematic supply chain stage in terms of GHG emissions and profit generation, indicating incongruities that constrain its sustainability. From this, it can be stated that both economically and environmentally, wheat production bears disproportionate costs.

Previous studies report that global GHG emissions from croplands (25% of agricultural GHGs) are second only to emissions from livestock production (Reay et al., 2012). Moreover, across the whole supply chain from cultivation to food production, crop cultivation may be the primary contributor to GHG emissions, contributing as much as 50–80% (Garnett, 2011; Jensen and Arlbjørn, 2014). In the present study, 77% of GHG emissions were produced during wheat cultivation, which is similar to previous findings (Fig. 5). In wheat cultivation, the use of chemical fertilizer alone accounted for 56.4% of GHG emissions. Overuse of chemical fertilizers, especially N, is one of the largest sources of carbon emissions (Tilman et al., 2011).

Low NUE can explain high GHG emissions in wheat cultivation. Compared with developed countries, NUE in major Chinese crops is reportedly less than 45%, while in the United States, it is as much as 70% (Zhang et al., 2015). For the present study, NUE in wheat production was 47.2% (Fig. 3). In China, pursuing high grain yield by chemical fertilizer is widely used practice (Jiao et al., 2019). On the North China Plain, twice as much chemical fertilizer has been applied than has been recovered from crops, a value that is much higher than the maximum use levels recommended by scientists (180–230 kg N ha^−1^) (Vitousek et al., 2009; Liu et al., 2016).

With such excessive fertilizer use, soil N availability in the root zone exceeds the requirements of wheat growth, resulting in substantial fluxes of N losses in the root zone and threshold responses due to the high magnitude of the N input (Reay et al., 2012). In addition, as a consequence of labor shortages and limited mechanization, as much as two-thirds of farmers in China typically apply all fertilizers in a single dose at the sowing stage (Chen et al., 2014). Under these conditions, NO_3_-leaching and N_2_O loss are even more likely. In the present study, chemical N input was as much as 304 kg ha^−1^ for 7.5 t ha^−1^ of wheat grain, leaving 140 kg of N ha^−1^ in the croplands (Fig. 4). Up to 7.5 kg of NH_3_ was emitted from croplands (Fig. 2). From previous research, the maximum N surplus estimate in wheat production was 60 kg ha^−1^ on the North China Plain (Ying et al., 2017; Zhang et al., 2018), which exceeds the critical holding capacity threshold in the root zone and would result in excessive GHG emissions.

Besides serious environmental effects, low NUE impairs profit due to smallholder mismanagement of wheat cultivations (Blasi et al., 2016). For example, smallholder-dominated wheat production on the North China Plain is constrained by the high heterogeneity of field conditions and lack of effective agronomy information for farmers, which has resulted in low technological practice innovation (Cui et al., 2010). This, in turn, has resulted in low wheat yields from excessive chemical fertilizer use and labor input. From the food supply chain perspective, to produce the same amount of steamed bread, more land, chemical fertilizer, and machinery will be needed, thus increasing the cost of the steamed bread. In the present study, to produce 1214 kg of grain for 1220 kg of steamed bread, 389 USD was needed, including 221 USD for land preparation, 70 USD for chemical fertilizer, and 99 USD for land preparation and harvesting machinery (Fig. S3). The net profit was only 39 USD, therefore the BCR was only 1.1 (Fig. 6b), which was much lower than in the UK and Germany (Jensen and Arlbjørn, 2014). It means that very little profit was obtained from the food supply chain by smallholders.

Compared with wheat cultivation, flour processing and steamed bread production was knowledge intensive. The N losses were minimal and NUE was up to 95% (Fig. 3), which was close to values determined for conventional bakeries in the UK and Germany, due to similar pretreatment technologies (Jensen and Arlbjørn, 2014). GHG emissions were only 23.1% of the whole supply chain, and the economic benefit was as much as 463 USD, representing 92.4% of the whole supply chain benefit (Fig. 6). However, a smart and responsive network device will be needed to update the manufacturing systems for the sustainability of the supply chain (Kliestik et al., 2020). Therefore, more attention should be given to improving wheat cultivation by smallholders to reduce the incongruities between NUE, GHG emissions, and economic benefits.

### 4.2 Local actions for decoupling GHG emissions and economic benefit by improving NUE

Implications of this research for improving the sustainability of the wheat supply chain mainly highlights the contribution of wheat cultivation by the different farmer practices: FP, practices by STB farmers, and optimal solution practices demonstrated under OPT scenarios. Compared with FP, practices by STB farmers increased net profit by 1.3 times and reduced GHG emissions by 16% across the entire supply chain. In even greater contrast, compared to FP, practices by OPT farms increased net profit by 2.6 times and reduced GHG emissions by 39% (Fig. 5, Fig. 6). NUE was improved to 62% and 83% for STB farmers and OPT scenarios, respectively, as compared to that for FP (Fig. 3). The majority of improvements were attributable to improved NUE, which yielded both economic and environmental benefits, indicating great potential for improving the sustainability of the supply chain through scientist–farmer engagement in wheat production and integration of all stages.

In the present study, compared with FP, practices by STB farmers, GHG emissions were reduced by 21% and profits increased 1.6 times for wheat cultivation. OPT scenarios reduced GHG emissions by 48% and increased profits by 1.7 times (Fig. 5, Fig. 6). Both STB farmers and OPT scenarios employed adaptive technologies to improve the sustainability of wheat production. First, the seeding rate was optimized to ensure high-yield wheat production (Table S1; Figs. 6 and S4) based on the optimal North China Plain seeding rate of 200 kg seed ha^−1^ to maximize the wheat spike number and avoid lodging at harvest (Lu et al., 2016). Second, both STB farmers and OPT scenarios reduced excessive chemical N fertilizer and maintained an optimal N concentration in the root zone based on the demands of high-yielding wheat (Zhang et al., 2013). Typically, for an 8 t ha^−1^ wheat yield, 250 kg ha^−1^ N can meet crop demand without a large amount of chemical N loss (Ying et al., 2017). In the present study, 258 kg ha^−1^ of chemical N fertilizer was used by STB farmers (Table S1, and Fig. S4), reducing the concentration of environmental N, such as N deposition and N in irrigation water. Overall, N loss for STB farmers and OPT scenarios was reduced by 38% and 70%, respectively (Fig. 4).

Improving NUE through adaptive technologies reduced GHG emissions and increased profitability. Owing to these adaptive technologies, the cost of agricultural inputs, including land preparation and chemical fertilizer, was reduced greatly in practices by STB farmers (Fig. S3). Meanwhile, wheat yield also increased significantly (Table S1). These results are consistent with previous studies on rice production (Stuart et al., 2017), which found that improved agronomic practices increased farmer incomes and reduced negative environmental effects.

The STB farmers have put the experimental findings into practice by improving technology adaptation. In this study, STB farmers used evidence-based wheat production, with 95% of STB farmers adopting reduced seeding rates and 74% adopting reduced chemical N fertilizer applications (Fig. 7a and b). This resulted in reduced total wheat production costs, hence improving supply chain sustainability. Empowering smallholders with adaptive technologies through participatory research is an effective approach to improve the income of wheat production, as scientists and smallholders work together to develop an appropriate innovative culture for continuous technology innovation, thereby stimulating the smallholders’ creativity (Zhang et al., 2016). In this way, the STB farmers not only received information and comprehended the process requirements, but also understood why the process was necessary (Huang et al., 2015). Farmers can use the adaptive technologies in their own field plots and also transfer technologies to neighbors (Jiang et al., 2020). With this approach, smallholders are equipped with knowledge of sustainable wheat production, and also become capable of mastering the required technological skills.

Food supply chains link producers, processors, markets, distributors, consumers, and smallholders (Blasi et al., 2016). Therefore, improving the income of smallholders is important to enhance the overall supply chain sustainability. In this research, for the whole wheat supply chain, smallholders' creativity was shown to be further stimulated, suggesting an increase in the demand for technology improvements to achieve a more sustainable supply chain (Jiang et al., 2020). Multidisciplinary and systematic knowledge linking environmental costs and economic benefits will be needed to improve the overall supply chain sustainability. From this study, the demonstration of the smallholder engagement approach has pushed smallholders to conduct technology improvements based on the supply chain sustainability demand. With this approach, wheat production can be optimized based on consumer demand and profit can be improved by matching production and consumption (Poore and Nemecek, 2018). Compared with FP, practices for the OPT scenarios reduced GHG emissions by 39% and increased profit by 2.6 times due to whole-meal bread production based on consumers’ demand (Fig. 5, Fig. 6). This indicates that the involvement of smallholders in the STB platform enables their adoption of enhanced management practices; moreover, engaging smallholders in the supply chain further stimulates their creativity in terms of contributing to supply chain sustainability.

### 4.3 Policy implications

**Fig. 8:**
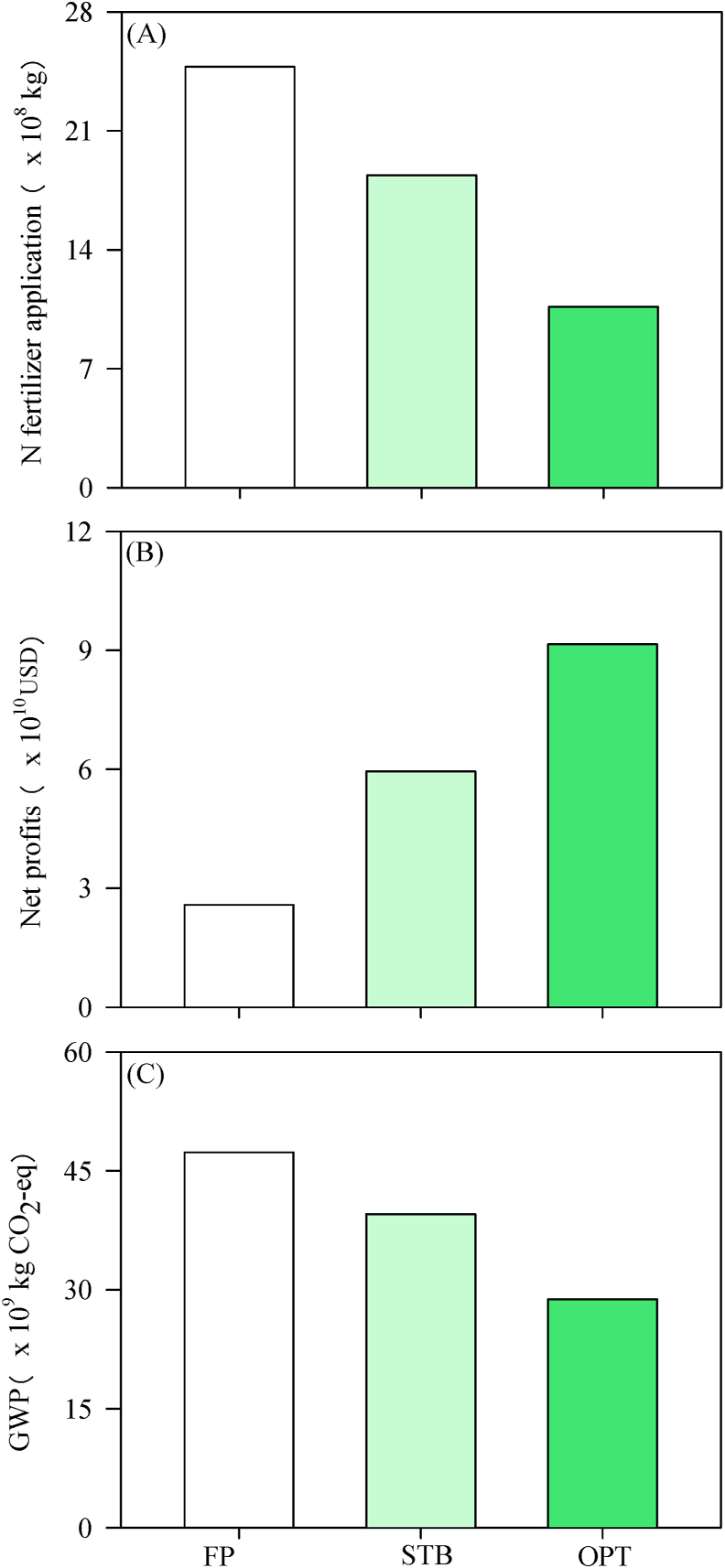
National-level chemical N fertilizer use (A), net profits (B), and GWP (C) for the wheat supply chain under the different groups: FP, STB, and OPT.

Supply chain sustainability cannot be achieved without cross-sector integration, including that of governments, enterprises, and knowledge providers (Horton et al., 2016). Considering the complexities of supply chains and vulnerability of smallholders, relying solely on farmers' social responsibility to achieve supply chain sustainability is unfeasible. Polices should therefore be focused on improving the economic attractiveness of supply chain sustainability, thereby attracting more smallholders’ involvement and further encouraging their contributions to GHG emission mitigation and resource conservation. If smallholder farmers across China follow best practices suggested by this research, up to 6.4 × 10^8^ kg of chemical N fertilizer could be saved and 7.8 × 10^9^ kg of GHG emissions could be prevented (Fig. 8). Local and regional governments should integrate more resources to empower smallholders by organizing cooperatives, creating market links, and providing developmental support at the farmer–community level. This would enable the supply chain to balance the multiple objectives of improving NUE, reducing GHG emissions, and increasing economic benefits (Fanzo, 2017). Findings of this study provide a baseline for future research exploring the working mechanisms linking smallholders with markets on an elemental level to improve overall food supply chain sustainability.

### 4.4 Limitations and uncertainties

We acknowledge that the N flow data used in the SFA model is subject to uncertainties, potentially impairing the robustness of the results to some extent. This is because the data on wheat production, flour processing, and bread production were obtained through surveys. More case studies should have been investigated in the study to reduce these uncertainties. Furthermore, to improve the accuracy of the data, real-time monitoring of N flow in wheat production is necessary. Besides, P should also be considered for assessing the sustainability of food supply chains in future studies. Moreover, due to a lack of data availability, some processes were not considered within the system boundaries, such as fertilizer, flour, bread storage, and transportation. Previous studies have showed that GHG emissions and economic costs from these stages only accounted for less than 5% of total GHGs (Espinoza-Orias et al., 2011; Osei-Owusu et al., 2019). Compared with the primary processes in the research, these are often considered negligible for environment risk and economic benefits. Therefore, we placed more effort on the sustainability of t primary processes.

## 5 Conclusions

As primary material providers, smallholders play a great role in the sustainability of the wheat supply chain in China. To produce 1220 kg steamed bread to meet the daily consumption needs of 10,000 people, compared with conventional farmer practices, STB farmers reduced GHG emissions by 16%, improved NUE by 32%, and increased economic benefits by 30% through adaptive technology use during wheat production. The OPT scenarios demonstrated a further reduction in GHG emissions (39%) and increases in NUE (76%) and economic benefits (2.6-times) through the integration all individual stages of the supply chain as a whole. Findings show that both environmental and economic objectives were simultaneously addressed through enhanced adaptive management practices (integrated nutrient management strategy) and engagement in food supply chains by empowering smallholders, demonstrating effective approaches to improve the overall sustainability of the entire food supply chain. Further research should be focused on working mechanisms of these approaches through multi-stakeholder engagement on the ground.

## CRediT authorship contribution statement

**Lijuan Deng:** Conceptualization, Methodology, Software, Data curation, Visualization, Investigation, Software, Validation, Writing – original draft. **Hongyan Zhang:** Data curation, Writing – original draft, Software, Validation. **Wenqi Ma:** Supervision. **Annah Zhu:** Data curation, Writing – original draft. **Chong Wang and Fusuo Zhang:** Supervision. **Xiaoqiang Jiao:** Writing – review & editing.

## Declaration of competing interest

The authors declare that they have no known competing financial interests or personal relationships that could have appeared to influence the work reported in this paper.
