# Supplementary material for: Improving the sustainability of the wheat supply chain through multi-stakeholder engagement
Source: J Clean Prod. 2021 Oct 25;321:128837. doi: 10.1016/j.jclepro.2021.128837 (PMC8527860; doi:10.1016/j.jclepro.2021.128837)
Supplement: Multimedia component 1 [file mmc1.doc]

Fig. S1 System boundaries in the wheat to steamed bread supply chain in Quzhou County. Black, green, blue and red arrows represent the inputs, products, byproducts and undesired outcomes, respectively.

Fig. S2 Distribution of surveyed smallholders, STB farmers and long-term field experiments (a) and enterprises engaged in flour production and steamed bread production (b) in Quzhou County.


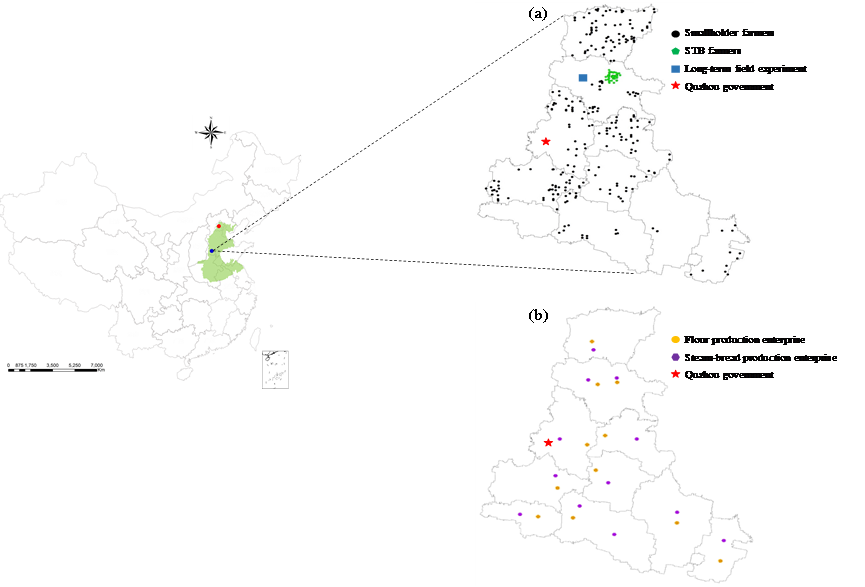


Fig. S3 Flowchart of research methodology

Fig. S4 Costs for producing steamed bread in each component of the supply chain under different agronomic systems: typical farmer practices in the region (FP), farmers engaged in STB programs (STB), and optimal solutions based on experimental field testing (OPT). Values were calculated based on the daily consumption needs of 10,000 people (1,220 kg of steamed bread).


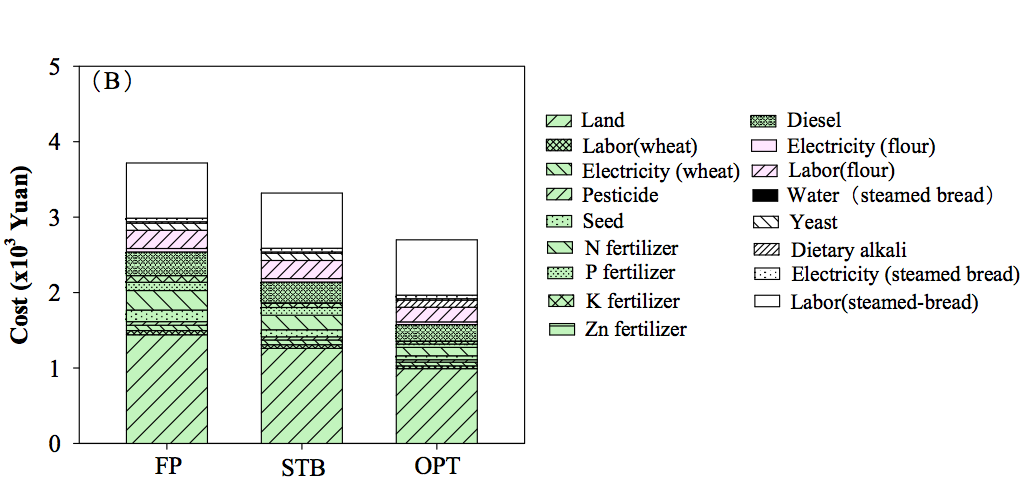


Fig. S5 Sowing rate (A), N application rate (B), and wheat yield (C) under current farmer practice (FP) and STB farmers in Quzhou County.


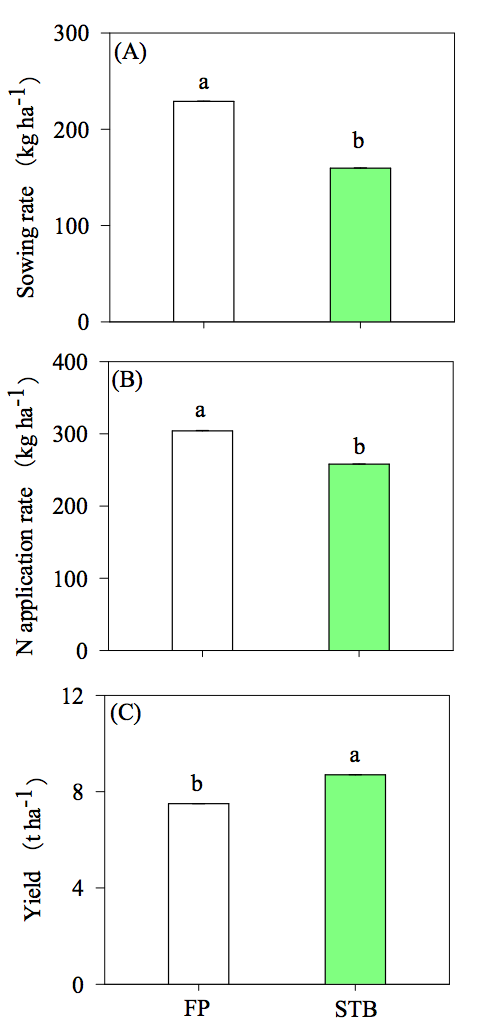


Table S1 Supply chain stages, their component processes and the input of materials for each stage in the wheat to steamed bread supply chain under different agronomic systems: typical farmer practices in the region (FP), farmers engaged in STB programs (STB), and optimal solutions based on experimental field testing (OPT). Values were calculated based on the daily consumption needs of 10,000 people (1,220 kg of steamed bread).


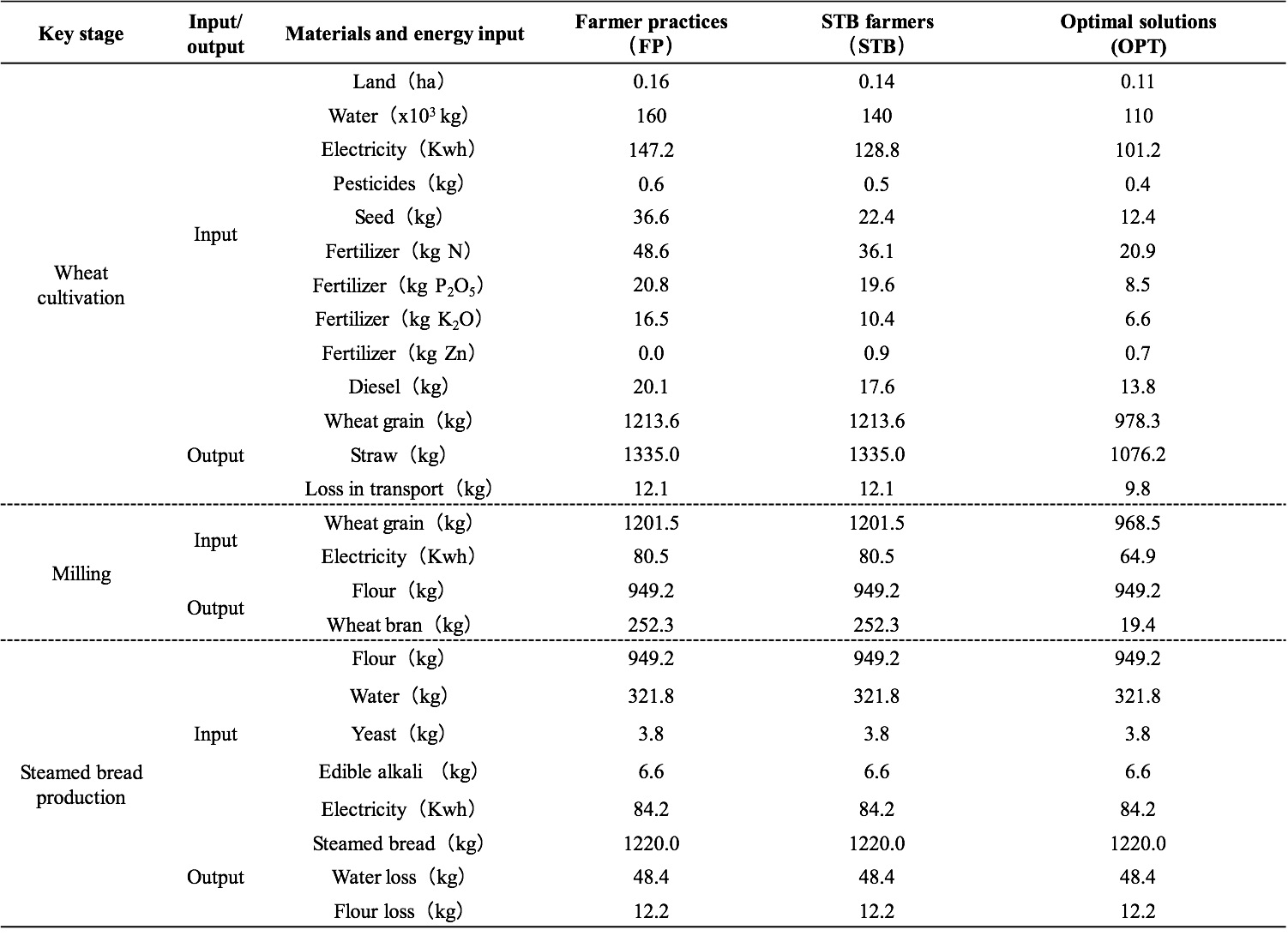


Table S2 Description and summary statistics for net profits (USD) under different farm types (FP, STB and OPT) used in the study. Numbers (N), mean and range (minimum to maximum), were presented in the brackets.

| Items | FP | | STB | | OPT | |
| --- | --- | --- | --- | --- | --- | --- |
|  | N | Value (USD) | N | Value (USD) | N | Value (USD) |
| Wheat cultivation | 265 | 38.8 (21.1, 49.5) | 59 | 99.9 (65.4, 120.1) | 1 | 103.5 (103.5, 103.5) |
| Milling | 10 | 5.2 (4.1, 6.4) | 10 | 5.2 (4.1, 6.4) | 10 | 30.7 (28.1, 39.4) |
| Steam-bread production | 10 | 463.1 (367.1, 601.4) | 10 | 1063.1 (800.7, 1300.9) | 10 | 1663.0 (1102.7, 1904.2) |
